# Modeling the Future Distribution of *Trifolium repens* L. in China: A MaxEnt Approach Under Climate Change Scenarios

**DOI:** 10.3390/biology14111608

**Published:** 2025-11-17

**Authors:** Haojun Wang, Qilin Liu, Jinyu Shen, Jiayu Ding, Yu Zeng, Zixin Zhou, Xiangrong Yan, Jianbo Zhang, Xiao Ma, Qingqing Yu, Yanli Xiong, Yi Xiong

**Affiliations:** 1 College of Grassland Science and Technology, Sichuan Agricultural University, Chengdu 611130, China; f2543471665@126.com (H.W.);; 2 Sichuan Academy of Grassland Sciences, Chengdu 611731, China

**Keywords:** *Trifolium repens* L., MaxEnt, niche, climate variables

## Abstract

*Trifolium repens* L. is a protein-rich, versatile Leguminous lawn plant that is widely distributed across global temperate and subtropical regions. As an invasive species originating in Europe, its distribution in China extends from Xinjiang in the West to Taiwan and the Yangtze River Delta in the East, and is widespread throughout Northeast and Central China. However, in recent years, the distribution pattern of *T. repens* has become increasingly patchy and irregular. Therefore, unraveling the potential distribution and key environmental drivers of *T. repens* is critical for understanding its ecological role. This study utilized current species distribution data of *T. repens* and employed the MaxEnt model to simulate its potentially suitable niches across present and future climate scenarios (SSP1-2.6, SSP2-4.5, SSP3-7.0, and SSP5-8.5) in China. This study identified Bio2 (mean diurnal temperature range) and Bio14 (precipitation of driest month) as the key drivers shaping the distribution of *T. repens*. Its current suitable habitats are primarily concentrated in the coastal, central, and Taiwan regions of China. Under future climates, these areas are projected to contract overall and shift toward lower latitudes and higher longitudes, with substantial suitable areas remaining only in the Eastern, Southern, and Taiwan regions. This study quantitatively assessed the ecological niche breadth of *T. repens* and its future spatial distribution under climate change, thereby laying a theoretical foundation for describing the ecological characteristics of this invasive species, conducting monitoring, and implementing further invasion risk management.

## Simple Summary

A critical knowledge gap exists regarding the potential distribution and future geographic niche shifts in the invasive species *Trifolium repens* L. in China. To address this gap—while explicitly recognizing the species’ capacity for ecological niche drift and human—mediated dispersal—we applied a parameters—optimized MaxEnt model to simulate its ecological niche distribution under current and future climates. The results reveal that the species’ currently suitable habitats are primarily concentrated in Southeastern coastal regions and Taiwan in China. Under future climate conditions, the suitable habitats are projected to undergo a notable contraction in total area, accompanied by a directional shift toward lower latitudes and elevations. The key climatic drivers identified were Bio2 (mean diurnal temperature range) and Bio14 (precipitation of driest month), which critically regulate its distribution limits. By integrating climatic constraints with dispersal—related distribution mechanisms, this study provides a spatially explicit basis for monitoring the spread of *T. repens* and supports the early—warning management of its invasion risk under climate change.

## 1. Introduction

*Trifolium repens* L. (white clover) is a perennial legume of global importance, valued not only as a high—quality forage crop but also for its roles in nitrogen fixation, soil conservation, and ecosystem restoration [1]. This species is native to Europe, and it has been introduced worldwide and is now widely naturalized in China, where it is utilized in agricultural, horticultural, and soil—improvement contexts [2,3]. However, its biological traits—including high seed production, strong vegetative spread, and broad environmental tolerance—have also made it a successful exotic invasive plant in China, where it can alter native plant communities and ecosystem processes [4]. Recent genomic studies reveal that during its global spread, *T. repens* underwent rapid parallel climate adaptation driven by large—effect structural variants, maintained through repeated introductions and gene flow [5]. This evolutionary agility highlights its potential to expand into new regions, complicating efforts to predict and manage its future distribution. However, existing research has largely focused on agronomic traits, stress tolerance, and medicinal properties [6,7], leaving a critical gap in understanding how its documented adaptive mechanisms translate into macro—scale distribution patterns under climate change. This gap is particularly concerning given its invasive potential and the increasing frequency of extreme climate events, which may interact to accelerate its spread or ecological impact [8].

Environmental variables are known to modulate plant community structure and drive niche shifts by altering spatial climatic conditions [9], highlighting their crucial role in shaping species’ developmental trajectories and ecological niche dynamics. Specifically, lower temperatures often prolong leaf longevity, enhancing competitive advantage through improved resource-use efficiency [10]. Altitudinal variation further influences community-weighted mean traits in both native and exotic plant assemblages, leading to shifts in niche width [11]. Additional factors such as soil properties, topography, and slope have also been shown to significantly affect species’ niche characteristics [12]. Therefore, investigating the environmental drivers of niche distribution is essential for predicting future species habitat shifts and informing conservation strategies under climate change. In studies of forecasting current and future species niche distributions, many niche models, including Generalized Additive Model (GAM), Boosted Regression Trees (BRT), and Random Forest (RF), and other models, all require both species presence and absence data [13,14]. In contrast, the MaxEnt model relies solely on species presence data and has proven effectiveness in extracting and preserving reliable information from distribution records [15]. The MaxEnt framework offers a versatile and customizable approach that enhances prediction accuracy while providing ecologically interpretable outputs [16,17]. This approach allowed the reconstruction of spatiotemporal distribution diagrams across multiple species and regions, clearly representing the mechanistic relationships between species and their environmental responses, and providing an effective tool for surveying and utilizing species resources [18,19]. However, despite being one of the most widely cultivated species of *Trifolium*, with more than 250 documented cases of cultivation, *T. repens* exhibits a relatively narrow ecological niche breadth, particularly with respect to temperature and precipitation [20]. These constraints markedly limit its autonomous adaptive capacity under climate change, thereby increasing the risk of habitat contraction and fragmentation in the face of increasingly frequent extreme climate events [8].

Invasive species that exhibit high dispersal ability and ecological drift—such as *T. repens*—frequently demonstrate non-equilibrium dynamics in novel environments. This phenomenon challenges the conventional assumptions of niche models [11]. However, the MaxEnt model has been widely and successfully applied in predicting invasion trajectories for such species (e.g., *Eriochloa villosa* [21], *Phytolacca americana* [22]), because it relies solely on occurrence data and can handle non-equilibrium distributions while identifying potential habitat suitability under changing climates [15,16,23]. As a globally cultivated species, *T. repens* exhibits high sensitivity to changes in water and temperature conditions due to its ecological adaptability. While such bioclimatic constraints would typically limit autonomous adaptation under climate change [24], its actual distribution has been substantially facilitated by human-mediated dispersal and rapid evolutionary shifts—traits characteristic of species with high ecological drift. This divergence between its fundamental niche and realized distribution makes *T. repens* a compelling candidate for MaxEnt modeling, which can help delineate the potential—rather than naturally constrained—spread of such invasive species, particularly under future climate scenarios.

This study applies the MaxEnt model to (1) identify the key environmental factors shaping the distribution of *T. repens* in China, (2) map its current and future suitable habitats under climate change scenarios, and (3) analyze its ecological niche breadth and spatial migration trends. By integrating genetic adaptation insights with spatial projection, we aim to provide a predictive framework that supports the proactive management of *T. repens*—not to promote its cultivation, but to anticipate its invasion pathways, mitigate habitat fragmentation risks, and inform strategic monitoring and control measures. The methodology established here also offers a transferable approach for studying other invasive species with similar ecological drift characteristics and niche vulnerabilities under global climate change.

## 2. Materials and Methods

### 2.1. Acquisition of Species Occurrence Data

To ensure comprehensive and reliable occurrence data, this study utilized two authoritative biodiversity databases: the Chinese Virtual Herbarium (CVH, https://www.cvh.ac.cn (accessed on 12 January 2025)) and the Global Biodiversity Information Facility (GBIF, https://doi.org/10.15468/dl.242anh (accessed on 7 April 2025) [25]). A total of 2160 occurrence records of the species, encompassing precise temporal and geographical details, were initially collected across China. To ensure data quality, records lacking coordinates or with ambiguous descriptions were georeferenced using Google Maps. Subsequently, the data were subjected to a rigorous cleansing process, during which entries exhibiting ambiguity in their location, redundancy due to duplication, or spatial redundancy were removed. Specifically, we used the SDMtools package in R to retain only one record per 5 km × 5 km grid, thereby minimizing spatial autocorrelation [26]. The final distribution dataset for *T. repens* in China comprised 481 rigorously validated screened occurrence records (Figure S1). This dataset was structured in a three-column CSV format (species name, longitude, and latitude in decimal degrees) and constituted the primary input for the MaxEnt model to predict both current and future species distributions [15].

### 2.2. Collection and Preprocessing of Environmental Variables

#### 2.2.1. Acquisition of Current Environmental Climate Variables

In this study, 19 bio-climatic variables spanning 1970–2000, along with 48 months of mean temperature and precipitation data, were obtained from the WorldClim database (https://worldclim.org (accessed on 11 January 2025)). Additional topographic variables —Altitude, Slope, and Aspect—were sourced from the RESDC (https://www.resdc.cn (accessed on 18 January 2025)). Details of the 22 environmental variables are provided in Table S1. A Jackknife test was used to preliminarily evaluate variable importance through single-variable modeling. The contribution percentage and permutation importance of the variables are calculated (Table S2), which will provide a foundation for the subsequent selection of variables [26,27].

#### 2.2.2. Accessing Future Climate Data

To predict the niche shifts under various future climate scenarios, future biological climate data were obtained from the Climate Change, Agricultural and Food Security (CCAFS, https://ccafs-climate.org (accessed on 19 February 2025)) database. This data was derived from an ensemble of the Coupled Model Inter-comparison Project Phase 6 (CMIP 6) in a multi-model context. In order to reduce the uncertainty in single-model outputs, four SSP–RCP scenarios (SSP1-2.6, SSP2-4.5, SSP3-7.0, and SSP5-8.5) were selected from the mainstream ACCESS-ESM1-5 BCC-CSM2-MR CanESM5 data source [28]. These scenarios encompass a range of greenhouse gas emission intensities [27]. Simultaneously, we obtained downscaling and bias correction data for climate factors in ISIMIP3b (v1.1) (https://www.isimip.org/gettingstarted/isimip3b-bias-adjustment/ (accessed on 25 January 2025)). This approach effectively reduced systematic biases of the GCMs data sources. Furthermore, each SSP-RCP scenario was subdivided into four time periods (Current period, 2030–2049, 2050–2069, 2070–2090), which correspond to the standard time periods employed in CMIP6.

#### 2.2.3. Spatial Standardization and Data Formatting

All environmental variables were processed in ArcGIS 10.8 to secure the spatial consistency. The procedures are as follows: (1) Standardized spatial resolution, whereby all variables were resampled to 2.5 arc-minutes (approximately 4.1 km by 4.1 km), which could keep a balance between computational efficiency and prediction accuracy. (2) Formatted conversion, whereby all preprocessed variables were converted to ASCII format to conveniently facilitate subsequent model input [26]. Additionally, the standard Chinese maps were obtained from the National Standard Map Service System (http://bzdt.ch.mnr.gov.cn, (accessed on 19 January 2025)), which served as the spatial analysis and visualization of *T. repens* distribution prediction results within the geographical boundaries of China [26].

### 2.3. Correlation Screening of Environmental Variables

#### 2.3.1. Pearson Correlation Coefficient Calculation

The Pearson correlation coefficient (r) was applied to quantify the linear relationship between pairs of raw environmental variables [29]. The attribute values of each environmental variable were extracted using ArcGIS 10.8, with the data extracted to raster format. The pairwise correlation values between variables were calculated based on IBM SPSS Statistics 26 [30].

#### 2.3.2. Variable Selection Based on Correlation and Contribution

To mitigate overfitting resulting from multi-collinearity and autocorrelation among environmental variables, the correlation matrix among the 22 initial variables was analyzed using ENMtools [31]. Based on the preliminary modeling percent contribution of variables (Table S1), variable pairs with |r| > 0.85 were excluded to prioritize ecologically informative predictors, while those with |r| ≤ 0.85 were directly included [15] in the candidate set. After this screening, variables exhibiting both low correlation (|r| ≤ 0.85) and high contribution were retained [15]. This process yielded a final set of 9 environmental variables for the construction of MaxEnt model (Table 1).

### 2.4. Parameter Optimization of the Model

The predictive performance of the MaxEnt model is highly relative to two key parameters: Feature Classes (FC) and Regularization Multiplier (RM). The joint determination of these two parameters is instrumental for establishing the form of feature transformation and the complexity of the model, ensuring the prevention of overfitting [32]. In accordance with recent studies on optimizing SDM parameters, this study implemented parameter tuning using the ENMeval package in the R language [33]. The FC combination with ΔAICC = 0 (the lowest corrected Akaike Information Criterion) was selected as optimal parameters.

### 2.5. Model Evaluation and Identification of Dominant Environmental Variables

#### 2.5.1. Model Accuracy Assessment According to AUC and Tss

The AUC (the area under the receiver operating characteristic curve) and TSS (the true skill statistic) values are used to evaluate the accuracy of the model’s predictions [34,35]. The AUC and TSS values were calculated based on current species occurrences and background data, with model performance assessed via cross-validation. These metrics quantify the model’s ability to distinguish between presence locations and background points. Model prediction accuracy was rated based on the AUC value as follows: AUC > 0.9, “excellent”; 0.8 < AUC< 0.9, “good”; 0.7 < AUC < 0.8, “average”; 0.6 < AUC < 0.7, “poor”; AUC < 0.6, “fail”, indicating a prediction no better than random [36]. In this study, the training data were constructed with three-quarters of the distribution site occurrences of *T. repens*, while the remaining one-quarter of the data was imported for the test set. Therefore, the random AUC = 0.5 served as the reference baseline, and the repetitively gradient-based iterations were implemented to generate a series of association values. The ROC curve was plotted based on both the training and test sets, which visually demonstrated the accuracy of the model’s performance. Given the sensitivity of TSS both to false negatives and false positives in MaxEnt models, the accuracy of model predictions compared to actual conditions can be assessed based on the calculation of confusion matrices. In this study, the TSS value was employed to evaluate the model’s capacity to predict actual species distributions. According to Liu et al. [37], the TSS, sensitivity, and specificity values were derived from the model based on 10 iterative runs, and the model performance was identified based on TSS values: 0.6 < TSS < 0.8, “acceptable”; 0.8 < TSS, “excellent”.

#### 2.5.2. Dominant Variables Identification Based on Jackknife Examination

Dominant environmental variables were identified based on their permutation importance and contribution to the model. Specifically, a variable was designated as a dominant factor exclusively if it was positioned among the top six in terms of both contribution rate and permutation importance. Response curves were then visualized for these selected dominant variables [38,39]. This criterion has also been widely adopted in the field of SDM, which plays a crucial role in screening significant variables of species distribution [40]. Furthermore, a species presence threshold was commonly applied in ecological studies [41]. The model’s validation was conducted using a TSS-based threshold, whereby the continuous presence probability (0–1) was converted into a binary classification of “present” and “absent,” thereby enabling objective assessment of the model’s predictive accuracy [42]. Consequently, suitable habitats for *T. repens* were delineated as areas exhibiting the occurrence probability > 0.5. This threshold is a common component in niche modeling, serving to preliminarily distinguish highly suitable regions from other regions.

### 2.6. Generation of Prediction Distribution Maps

The median value obtained from multiple repeated simulations of the model under each scenario is adopted as the final prediction result. These median maps were converted to raster format and imported into ArcGIS. In ArcMap 10.8, fixed bins were utilized to reclassify China’s suitable habitat into 4 distinct suitability levels—unsuitable regions (*p* < 0.1), low-suitable regions (0.1 < *p* < 0.3), moderate-suitable regions (0.3 < *p* < 0.5), and high-suitable regions (*p* > 0.5) [43]. This multi-level suitability classification method will be applied to generate subsequent prediction maps of current and future suitable habitat distributions. This approach facilitated the visual representation of spatial pattern changes across different suitability levels and enabled the calculation of varying suitable habitat areas under future scenarios for each time period. In order to facilitate a more visual comparison of the shift trend in suitable habitats across future scenarios, the raster documents of the prediction maps for each time period were converted into vector format in ArcGiS. And the intersect tools were utilized to analyze, which are located within the Overlay toolbox. Eventually, the resulting vector file was then converted back to raster format for subsequent analysis.

In order to obtain the shift change in distribution of *T. repens* under various scenarios, this study employed optimized Maxent model parameters under SSP1-2.6, SSP2-4.5, SSP3-7.0, and SSP5-8.5 future scenarios. A projection was generated to simulate and visualize shifts in potentially suitable regions and the dynamics of the niche. In addition, this study quantified the specific expansion and contraction change in potential distribution for *T. repens* across China under four future scenarios compared with current conditions. The proportions of each suitability class were graphically visualized using a pie chart (Figure S2).

## 3. Results

### 3.1. Analysis of the Pearson Correlation for Variables

Pairwise correlations among the environmental variables revealed the considerable variation and complex interdependence, both among bioclimatic factors and between bioclimatic and topographic factors (Figure S3). Multiple variable pairs showed strong linear relationships, underscoring the necessity of preliminary variable screening. Notably, significant positive correlations are observed among key temperature- and precipitation-related variables (e.g., Bio1, Bio2, Bio14, Bio15). Topographic variables (altitude, aspect, slope) also exhibit substantial spatial heterogeneity in their correlations with bioclimatic factors. A pronounced negative correlations are identified between altitude and several temperature-related variables. Collectively, these results provide a critical baseline for elucidating the terrain and climate mechanisms governing the distribution of *T. repens*.

### 3.2. Importance Estimation of the Environmental Variables

Based on the contribution percent and permutation importance of environmental variables derived from the Maxent model (Table 2), six variables are eventually identified as the dominant factors of *T. repens* habitat suitability, including Bio2, Bio14, Bio15, Bio1, Bio4, and Altitude. These six variables of cumulative contribution rate collectively account for 96.9%, with the permutation importance values of 5.7, 6.2, 8.1, 8.6, 33.9, and 32.1. The output demonstrates that the important ecological values of these six variables are in exploring the ecological niche of *T. repens*.

### 3.3. Determination of Optimal FC and RM Based on ΔAICC

**Figure 1:**
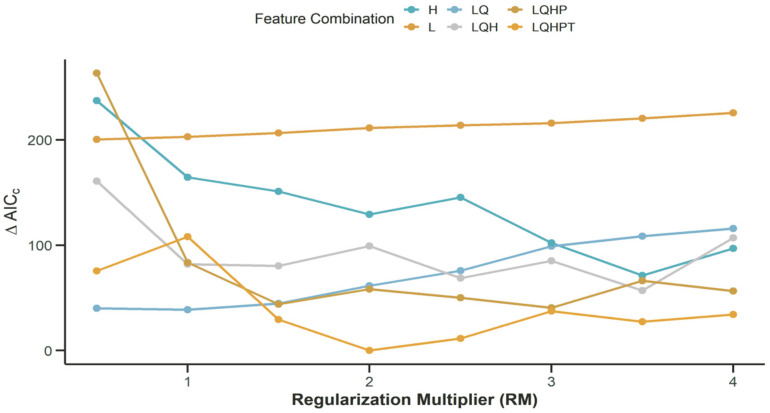
Model Parameter Optimization Diagram.

Testing various feature class (FC) combinations reveals distinct patterns in ΔAICC values. Specifically, FC = L results in low fluctuation but consistently high ΔAICC values. Both FC = H and FC = LQH exhibit a general downward trend in ΔAICC as the regularization multiplier (RM) increases. For FC = LQHP, ΔAICC initially rose but subsequently decreased and stabilized with increasing RM. Notably, none of the tested FC combinations achieve a ΔAICC value of zero across RM values from 0 to 4, with the exception of FC = LQHPT at RM = 2, where ΔAICC reached zero. Consequently, the combination of FC = LQHPT, RM = 2 is selected as the targeted parameters for model construction. This configuration improved the prediction accuracy and generalization ability of the Maxent model for the target species, *T. repens* (Figure 1).

### 3.4. Evaluation of Predictive Accuracy Based on the AUC Curve and the TSS Value

**Figure 2:**
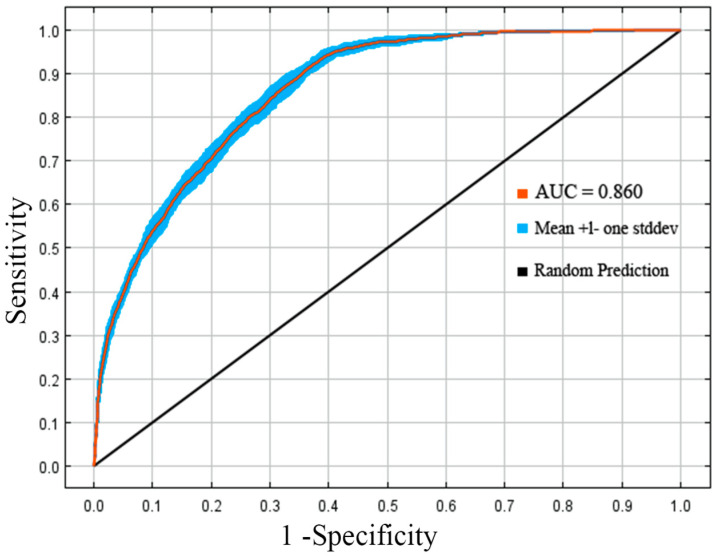
Model testing AUC curve.

In light of the aforementioned results, the parameter combination (FC = LQHPT, RM = 2) is selected to evaluate the model’s predictive accuracy in assessing the ecological suitability of *T. repens*. The findings indicate that the model demonstrates robust performance and high stability, with the AUC value of 0.860 attained under current climate conditions (Figure 2). This finding demonstrates the model’s robust and consistent predictive performance, supporting its use in forecasting species distribution under future climate scenarios. The evaluation of TSS (the True Skill Statistic) showed a mean value of 0.865 for the model (Table S3). Both TSS and AUC demonstrate that the model accurately identifies species presence regions while correctly excluding areas of absence. The findings demonstrate the high stability and retained precise predictive targeting of the MaxEnt model for *T. repens* niche simulation under complex background changes in environmental conditions.

### 3.5. Analysis of Dominant Variables and Evaluation of Model Prediction Accuracy

**Figure 3:**
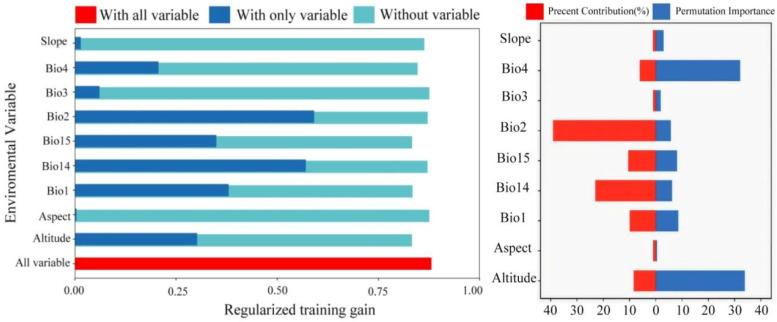
Analysis of dominant variables using jackknife cutting.

According to the jackknife test, the six most influential variables—based on their cumulative contribution percentages and permutation importance—are Bio2, Bio14, Bio1, Bio15, Altitude, and Bio4 (Figure 3). Response curves are generated to visualize the relationship between these dominant factors and the predicted probability of species occurrence. A thorough examination of these curves yielded the following optimal ecological ranges and amplitudes for each variable: Bio2 (range: 3.47–8.71; amplitude: 5.22), Bio14 (range: 16.16–222.20; amplitude: 164.51), Bio15 (ranges: 7.53–67.96 and 138.54–163.68; amplitude: 20.34), Bio1 (range: 12.84–25.30; amplitude: 16.84), Bio4 (range: 137.82–645.74; amplitude: 339.57), and Altitude (range: 0–137.87; amplitude: 137.87) (Figure S4). These results indicate that temperature, precipitation, and altitude are among the dominant environmental factors influencing the ecological adaptability and distribution of *T. repens*.

### 3.6. Distribution of Suitable Areas Under Four Climate Scenarios in China

**Figure 4:**
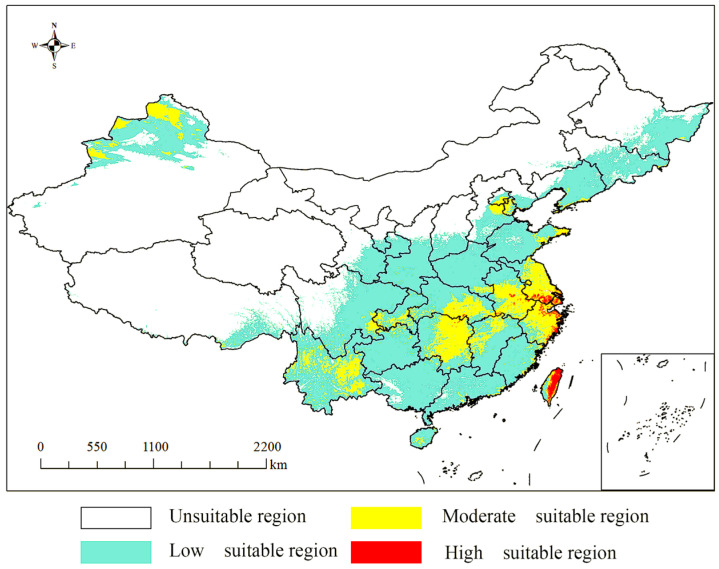
Prediction of the current potentially suitable distribution of *T. repens.*

**Figure 5:**
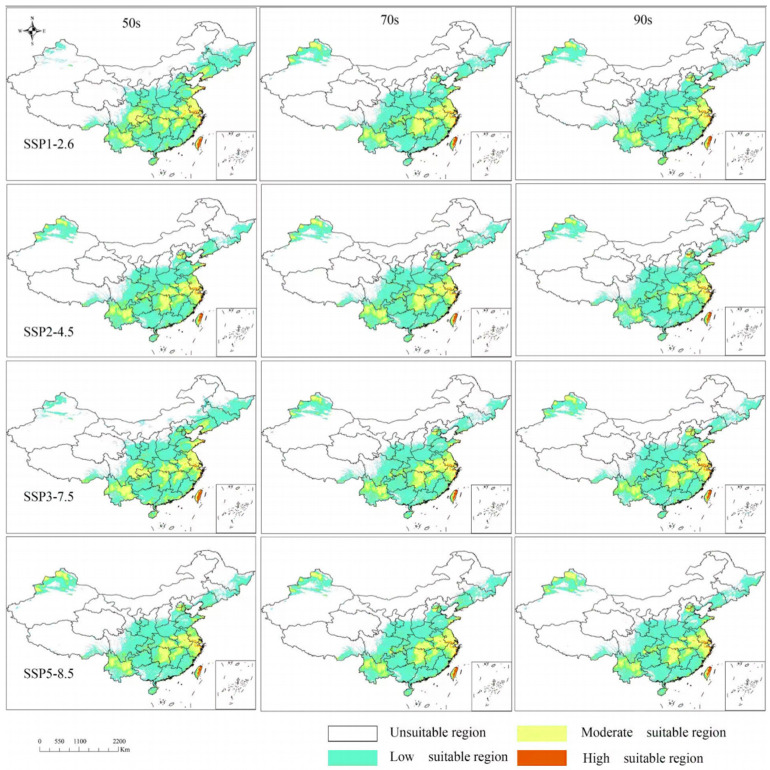
Predict the future potential suitable distribution under various scenarios.

As shown in Figure 4, the potentially suitable habitats for *T. repens* are primarily located in mountainous areas of North and Southeast China, the Sichuan Basin, the Eastern Yunnan–Guizhou Plateau, the Junggar Basin, and along the margins of basins and plateaus in the Eastern monsoon zone. Among these, the low-suitability areas are extensive, covering the North China Hills, Shandong Hills, Junggar Basin, Liaodong Hills, Sichuan Basin, and the Eastern Yunnan–Guizhou Plateau—regions that span the core hilly, basin, and plateau-edge landscapes of the Eastern monsoon region. The moderate-suitability areas occur as discrete, fragmented patches within the broader low-suitability zones, including the Shandong Hills, Southeastern Hills (e.g., Zhejiang and Fujian Hills), the Altay regional center, and other localized areas. The high-suitability areas are the most limited in extent, confined only to isolated locations such as the Southeastern coastal zones (e.g., in Zhejiang and Fujian) and the mountainous regions of Taiwan. The projected distribution of potentially suitable habitats under future climate scenarios is presented in Figure 5. The results suggest that future suitable areas in China will undergo a process characterized by “contraction in core zones, shift at marginal areas, and fragmentation in extreme habitats”. Distinct spatial changes are observed across the different suitability classes.

The total potential suitable distribution areas of *T. repens* in China under current conditions are estimated to be 3.6153 × 10^6^ km^2^ (Table 3 and Figure S2). However, the proportions of high suitable, moderate suitable, and low suitability areas, respectively, relative to the total suitable area, all exhibit annual variations in the four future climate scenarios. It is worth noting that the total potential suitable distribution areas of *T. repens* exhibit different degrees of contraction by the 2090s, with varying change regularities under each climate condition.

### 3.7. Dynamic Shifts in Distribution Under Various Climate Scenarios

**Figure 6:**
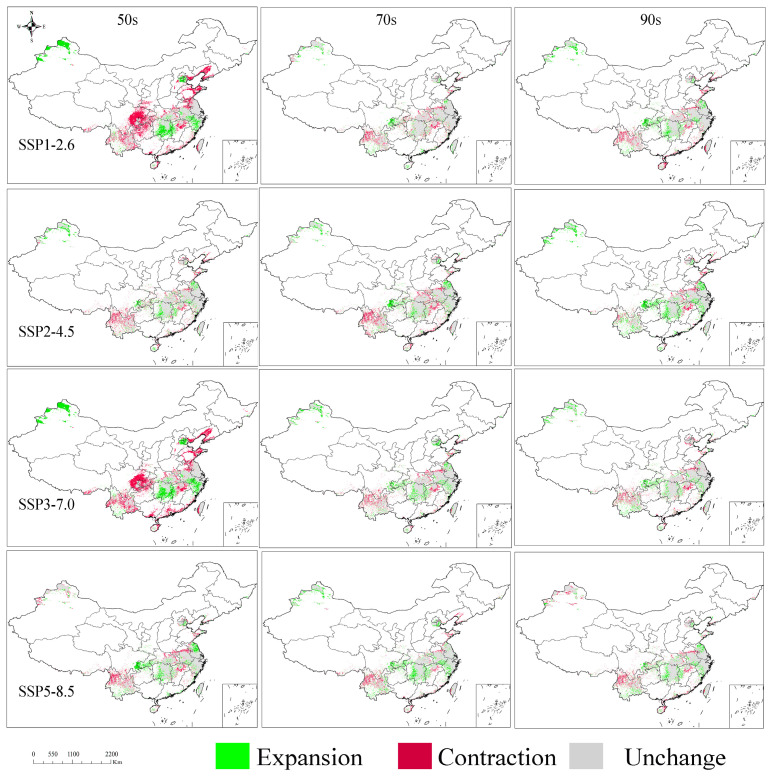
Dynamics of distribution range expansion and contraction of *T. repens* in the SSP1-2.6, SSP2-4.5, SSP3-7.0, SSP5-8.5 climate scenario during the 2050s, 2070s, and 2090s relative to current levels.

The projected change in the potentially suitable area for *T. repens* stabilizes gradually across all four future climate scenarios, with the variation in potentially suitable areas remaining below 0.6 × 10^6^ km^2^ under each scenario by the 2090s (Table 4 and Figure 6). It is noteworthy that the expansion of suitable areas shows a decreasing trend in Xinjiang (China) and the middle–lower Yangtze River basin. In contrast, areas exhibiting annual increases are mainly located in the border region between Sichuan and Chongqing, as well as Eastern Jiangsu Province. Conversely, the contraction of suitable areas shows a consistent decrease in Southwestern China, Southern Liaoning Province, Northern Shandong Province, Southern Tibet, and parts of Southern China, while a persistent increase was observed in central Xinjiang and Northern Yunnan. Overall, the entire potentially suitable area exhibits a general shift toward lower latitudes and higher longitudes, characterized by a concurrent expansion into Eastern and coastal regions of China.

## 4. Discussion

### 4.1. MaxEnt Model Selection, Optimization, and Performance Evaluation

The MaxEnt model has been demonstrated to offer distinct advantages over competing ecological niche models, such as ENFA and Occupancy [44]. This is primarily attributable to its capacity to harness species-specific attributes and generate reliable predictions with only presence data. The parameter optimization of the model is critical for enhancing the model’s generalization capability and ecological realism under diverse climate scenarios [37]. As demonstrated by Zhao et al. [33], the degree of species niche overlap is influenced by feature combination (FC), regularization multiplier (RM), and threshold selection, collectively. Thus, identifying an optimal parameter set is imperative for stabilizing the species-environment relationship and enhancing predictive accuracy.

In this study, we optimized the model parameters using the ENMeval 2.0 R package. The optimal configuration is determined to be the LQHPT feature combination with an RM value of 2, and these parameters were subsequently applied to model the suitable habitat distribution of *T. repens*. The tuned model achieved a ΔAICC value of 0, with AUC scores of 0.860 for the training set and 0.856 for the test set of the model, confirming high predictive precision and reliable performance under future climate scenarios. Additionally, the model’s TSS value of 0.865 substantiates its remarkable actual prediction matching capability. These results validate the stability and applicability of the MaxEnt model across varied environmental contexts for niche modeling. Furthermore, this model establishes a robust theoretical framework for assessing the impact of climate change on the habitat distribution of invasive species, thereby supporting fundamental ecological research.

### 4.2. Ecological Drivers and Current Distribution of T. repens

*T. repens* is a moisture-preferring legume with strong heat tolerance. The species’ capacity for resource acquisition and ecological competition is contingent upon temperature and precipitation conditions, which are also the predominant environmental factors enabling its invasion of China [7,20]. It is noteworthy that *T. repens* and rhizobia manifest a substantial nitrogen-ammonia interaction mechanism. This interspecific relationship exerts a substantial influence on the capacity to overcome habitat constraints of *T. repens* and compete for its ecological niche. Conditions conducive to its rapid growth include elevated mean temperatures, reduced diurnal temperature variation, and increased precipitation, which likely enhance the symbiotic efficiency with rhizobia and promote organic compound accumulation [45]. Therefore, an increase in the spatial niche dominance and potential distribution of *T. repens* may also be attributable to the presence of warm and humid regions [46]. Among 22 initial environmental variables assessed, six were identified as dominant factors (Bio2, Bio14, Bio15, Bio1, Bio4, and Altitude) based on the contribution rate, permutation importance, and autocorrelation among all variables. This finding serves to emphasize the pivotal role that hydrothermal conditions play in the adaptive growth of *T. repens*, a conclusion that is in accordance with the results reported in vegetation across Southwestern China [47]. Therefore, maintaining adequate water supplementation and appropriately elevated temperatures is maintained in order to guarantee stable nitrogen fixation efficiency, particularly during cool or dry periods [48,49].

The simulations of the MaxEnt model under current climatic conditions reveal substantial potentially suitable habitats for *T. repens* across China. These potentially suitable zones are primarily concentrated in Northern Xinjiang, Southern China, the Yangtze River Delta, Eastern coastal zones, and Taiwan. The environmental characteristics of these suitable areas align closely with the response curves of the six dominant variables, using a presence probability threshold of *p* ≥ 0.5. The derived optimal ecological values (e.g., Bio2 = 5.22, Bio14 = 164.51) closely match the hydro-climatic conditions of these regions. The most suitable habitats of *T. repens* are predominantly distributed in the Eastern coastal plains and areas East of the Taiwan Mountain Range. In these regions, the high temperature and low-latitude conditions correspond well with the species’ ecological niche. This correspondence is further explained by the species’ adaptations to drought and heat stress [49,50].

### 4.3. Future Niche Shifts and Heterogeneous Responses Under Climate Change

Despite its wide distribution and adaptability, *T. repens* exhibits heterogeneous responses in habitat suitability under future climate scenarios [51,52]. It is evident that ecological plasticity may be enhanced under prolonged arid conditions in the Eastern coastal and Taiwan regions, model projections indicate a substantial contraction of suitable areas in central China, Northern Xinjiang, and the Northeastern regions [53]. Conversely, expansions of high-, moderate-, and low-suitable areas are projected to varying degrees along the Eastern coastal regions and Taiwan. In essence, the potentially suitable habitats are transitioning toward subtropical and monsoonal regions over time. This phenomenon is consistent with the documented environmental preferences of this species [54]. These shifts may be attributed to the disruption of the critical hydrothermal synergy underpinning its resilience, compounded by additional drivers such as elevated CO_2_ levels. Another potential reason for these shifts is that suitable distribution areas may possess abundant soil nutrients or hydrological conditions, exhibiting exceptional ecological compatibility with *T. repens*’ growth. The aforementioned factors act in concert to determine its core competitive advantage as an invasive species within Chinese ecosystems [46,55]. Furthermore, the implications of the niche that emerged from interspecific relationships between *T. repens* and other species, such as rhizobia and insects, warrant consideration.

The projections under the four SSP scenarios demonstrate that varying trends of habitat expansion and contraction are attributable to differential changes in precipitation and temperature [56,57]. By the 2090s, an overall decline in habitat suitability is projected across all scenarios [58]. Under SSP1-2.6, a slight contraction in high-suitability areas contributes to a net decline. Under SSP2-4.5, the expansion in low-suitability areas is outweighed by significant losses in moderate- and high-suitability zones [59]. Notably, low-suitability regions are projected to decline sharply at an annual rate of 5654 km^2^. Moreover, the habitat exhibits an irregular trajectory under the SSP5-8.5 scenarios, characterized by an initial contraction, subsequent expansion, and subsequent renewed contraction, resulting in a marginal net reduction. In contrast to other scenarios, it is important to note that the total potentially suitable habitat expands from the 2050s to the 2090s. This trend is potentially driven by elevated carbon emissions, which may push hydrothermal conditions beyond the capacity of *T. repens*’ ecological tolerance [60,61]. Subsequent analysis confirms that topographic factors (e.g., aspect, slope) have also limited influence on niche shifts, whereas temperature and precipitation variables play a dominant role, consistent with their higher contribution in model outputs.

Compared to the impact of natural environmental conditions, the direct influence of human activities has also been exerted on the distribution of *T. repens*, with specific harvesting and conservation measures applied to the species serving as the primary drivers of this influence. Concurrently, the repercussions of anthropogenic activities on the environment have escalated annually, directly modifying the invasion rate of *T. repens* and decelerating its expansion trend across China.

### 4.4. Implications for Invasion Risk Management

The findings of this study provide critical insights for enhancing invasion risk assessment and early-warning systems for *T. repens* in China. The projected habitat shifts over the coming decades serve as a spatially explicit basis for prioritizing monitoring and intervention efforts in regions susceptible to further invasion. In particular, the identified areas of expanding suitability underscore the need for proactive measures by ecological management authorities to prevent further spread into ecologically sensitive zones. This study further substantiates the utility of the MaxEnt modeling framework in projecting the potential—rather than naturally constrained—distribution of invasive species characterized by ecological drift. By integrating occurrence data with climate projections, this approach effectively captures the dynamic interplay between environmental constraints and human-aided dispersal, offering a pragmatic tool for anticipating invasion pathways under non-equilibrium conditions. Instead of promoting its cultivation, these results underscore the necessity of restricting the deliberate introduction of *T. repens* into newly suitable habitats. It is imperative that management strategies prioritize the containment of its propagation in high-risk regions, particularly those that are anticipated to evolve into climatic refugia or invasion corridors. Furthermore, the methodology developed here can be adapted to other invasive species with similar dispersal traits, supporting the development of a more predictive and proactive biosecurity framework under global change.

## 5. Conclusions

This study employed MaxEnt model to simulate the environmental suitability and geographic invasion trends of *T. repens* under various future climate scenarios (SSP1-2.6 to SSP5-8.5). Results indicate that currently suitable habitats are mainly distributed across the North China Hills, Shandong Hills, Northern Southeast Hills, and Eastern Yunnan–Guizhou Plateau, covering a total area of approximately 3.6153 × 10^6^ km^2^ in China. The seasonal hydrothermal patterns are identified as the dominant climatic factor influencing the ecological niche shifts in *T. repens*. Under future climate scenarios, *T. repens* displays a notable degree of adaptability to climatic fluctuations. Core suitable habitats—such as the Northern Southeast Hills and the peripheries of the Sichuan Basin—remain stable across all SSP scenarios, although contraction risks persist.

## Figures and Tables

**Table 1:** Environmental variables applied to final model construction.

Environmental Variables	Description
Bio1	Mean Annual Temperature (°C)
Bio2	Mean Diurnal Range (Mean of monthly (max temp—min temp)) (°C)
Bio3	Thermal Uniformity (Bio2/Bio7(Temperature annual range)) (× 100) (%)
Bio4	Temperature Seasonality (Standard Deviation × 100)
Bio14	Precipitation of Driest Month (mm)
Bio15	Precipitation Seasonality (Coefficient of Variation) (%)
Altitude	Altitude (m)
Slope	Terrain Gradient (°)
Aspect	Orientation of the Slope

**Table 2:** Contribution rate of optimized environmental variables.

Environmental Variable	Percent Contribution (%)	Permutation Importance (%)
Bio2	39.1	5.7
Bio14	23	6.2
Bio15	10.5	8.1
Bio1	9.9	8.6
Altitude	8.4	33.9
Bio4	6.1	32.1
Slope	1.1	3
Bio3	1	1.9
Aspect	1	0.5

**Table 3:** Suitable distribution areas under present and future climate conditions.

	Decades	Low Suitable Region (10^4^ km^2^)	LOW/TOTAL (%)	Moderate Suitable Region (10^4^ km^2^)	MOD/TOTAL (%)	High Suitable Region (10^4^ km^2^)	HIGH/TOTAL (%)	Total (10^4^ km^2^)
-	current	284.68	78.74	73.09	20.22	3.76	1.04	361.53
SSP126	50s	278.19	74.11	92.21	24.57	4.97	1.32	375.37
	70s	274.96	77.58	76.09	21.47	3.36	0.95	354.41
	90s	275.80	78.54	71.91	20.48	3.45	0.98	351.16
SSP245	50s	274.13	77.63	75.27	21.32	3.72	1.05	353.11
	70s	273.94	77.73	75.32	21.37	3.18	0.90	352.44
	90s	279.34	80.94	62.51	18.11	3.27	0.95	345.12
SSP370	50s	293.03	75.96	88.67	22.98	4.09	1.06	385.79
	70s	272.26	78.40	71.77	20.67	3.25	0.94	347.28
	90s	270.45	77.96	72.19	20.81	4.26	1.23	346.90
SSP585	50s	273.71	78.36	72.80	20.84	2.77	0.79	349.28
	70s	287.77	79.93	68.90	19.14	3.38	0.94	360.04
	90s	279.43	78.29	74.19	20.79	3.28	0.92	356.91

Note: SSP1-2.6 represents a low emissions pathway; SSP2-4.5 represents an intermediate-low emissions pathway. SSP3-7.0 represents an intermediate-high emissions pathway; SSP5-8.5 represents a high emissions pathway.

**Table 4:** Change areas of suitable regions in different periods (Unit: 10^4^ km^2^).

Future Climatic Conditions	Decades	Expansion	Contraction	Unchanged	Total Area Change
SSP126	50s	20.77	40.36	54.64	61.14
	70s	7.98	10.74	67.42	18.72
	90s	12.43	11.20	62.99	23.63
SSP245	50s	11.06	12.80	64.36	23.86
	70s	11.82	13.67	63.56	25.49
	90s	18.40	7.62	56.98	26.02
SSP370	50s	22.82	38.95	52.58	61.78
	70s	12.73	10.36	62.69	23.10
	90s	10.98	10.05	64.46	21.03
SSP585	50s	13.60	12.57	61.76	26.17
	70s	14.61	10.02	60.78	24.64
	90s	10.73	11.09	64.72	21.82

Note: Total area change = Expansion + Contraction.

## Data Availability

The original contributions presented in this study are included in the article/Supplementary Materials. Further inquiries can be directed to the corresponding authors.

## Supplementary Materials

The following supporting information can be downloaded at: https://www.mdpi.com/article/10.3390/biology14111608/s1. Figure S1: Screening of current geographic occurrence records for *T. repens* in China; Figure S2: Fan-shaped chart of *T. repens*. suitable areas proportion in different periods in the future scenarios; Figure S3: Correlation Matrix Diagram of Climate Variables; Figure S4: Response curve of dominant environmental variables. Table S1: Description of the environmental variables selected for this study; Table S2: Contribution rate of initial environmental variables; Table S3: The TSS Values and Sensitivity Specificity Values of the MaxEnt model.
