# Supplementary material for: Modeling the Future Distribution of Trifolium repens L. in China: A MaxEnt Approach Under Climate Change Scenarios
Source: Biology (Basel). 2025 Nov 17;14(11):1608. doi: 10.3390/biology14111608 (PMC12650469; doi:10.3390/biology14111608)
Supplement: Supplementary file 1 [file biology-14-01608-s001.zip › Supplementary Material Figure S1.pdf]

Supplementary Material Figure

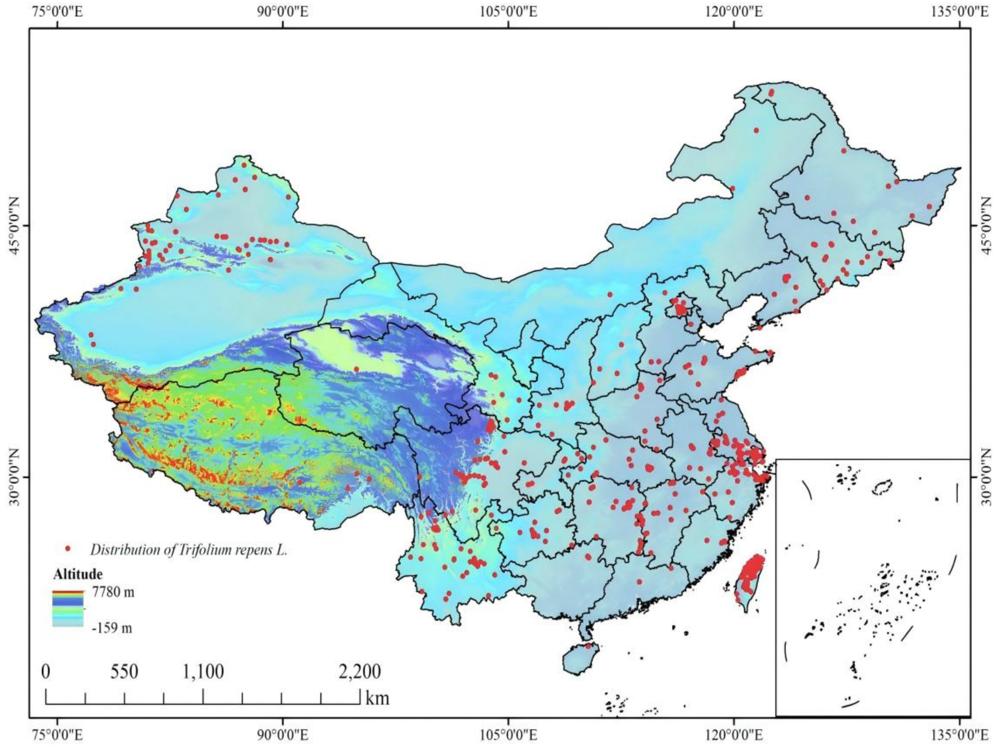

Figure. S1：Screening of current geographic occurrence records for *T. repens* in China.
