# Supplementary material for: Modeling the Future Distribution of Trifolium repens L. in China: A MaxEnt Approach Under Climate Change Scenarios
Source: Biology (Basel). 2025 Nov 17;14(11):1608. doi: 10.3390/biology14111608 (PMC12650469; doi:10.3390/biology14111608)
Supplement: Supplementary file 1 [file biology-14-01608-s001.zip › Supplementary Material Table S1.pdf]

Table S1 Description of the environmental variables selected for this study

Environmental variables	Description	Unit
Bio1	Mean Annual Temperature	°C
Bio2	Mean Diurnal Range (Mean of monthly (max temp - min temp))	°C
Bio3	Thermal Uniformity (BIO2/BIO7) (* 100)	°C
Bio4	Temperature Seasonality (Standard Deviation * 100)	°C
Bio5	Max Temperature of Warmest Month	°C
Bio6	Min Temperature of Coldest Month	°C
Bio7	Temperature Annual Range (BIO5 - BIO6)	—
Bio8	Mean Temperature of Wettest Quarter	°C
Bio9	Mean Temperature of Driest Quarter	°C
Bio10	Mean Temperature of Warmest Quarter	°C
Bio11	Mean Temperature of Coldest Quarter	°C
Bio12	Annual Precipitation	mm
Bio13	Precipitation of Wettest Month	mm
Bio14	Precipitation of Driest Month	mm
Bio15	Precipitation Seasonality (Coefficient of Variation)	mm
Bio16	Precipitation of Wettest Quarter	mm
Bio17	Precipitation of Driest Quarter	mm
Bio18	Precipitation of Warmest Quarter	—
Bio19	Precipitation of Coldest Quarter	—
Altitude	Altitude	m
Slope	Terrain Gradient	°
Aspect	Aspect of the Slope	—
