# Supplementary material for: Modeling the Future Distribution of Trifolium repens L. in China: A MaxEnt Approach Under Climate Change Scenarios
Source: Biology (Basel). 2025 Nov 17;14(11):1608. doi: 10.3390/biology14111608 (PMC12650469; doi:10.3390/biology14111608)
Supplement: Supplementary file 1 [file biology-14-01608-s001.zip › Supplementary Material Table S2.pdf]

Table S2 Contribution rate of initial environmental variables

Environmental variable	Percent contribution	Permutation importance
Bio14	31.7	1.8
Bio17	13.5	4.7
Altitude	9.4	22
Bio12	7.5	6.3
Bio15	7.1	8.4
Bio1	4.4	8.7
Bio4	4	11.5
Bio2	3.9	9.5
Slope	2.9	3.5
Bio7	2.3	0.5
Bio3	2.1	1.6
Bio13	2	3.5
Bio19	1.7	3
Bio6	1.5	2.6
Bio18	1.1	1.6
Bio9	1	2.8
Bio16	0.9	0.6
Bio10	0.8	3
Aspect	0.7	0.9
Bio11	0.6	1.2
Bio8	0.6	0.7
Bio5	0.4	1.6
