# Supplementary material for: Modeling the Future Distribution of Trifolium repens L. in China: A MaxEnt Approach Under Climate Change Scenarios
Source: Biology (Basel). 2025 Nov 17;14(11):1608. doi: 10.3390/biology14111608 (PMC12650469; doi:10.3390/biology14111608)
Supplement: Supplementary file 1 [file biology-14-01608-s001.zip › Supplyementary Materials Table S3.pdf]

Table S3 The TSS Values and Sensitivity Specificity Values of model

TSS	OverallAcc	Sensitivity	Specificity	Replicate
0.865931749460043	0.719009844212941	0.853131749460043	0.7128	0
0.865931749460043	0.685940934722355	0.88768898488121	0.6766	1
0.865931749460043	0.727229284144127	0.838012958963283	0.7221	2
0.865931749460043	0.715569148427793	0.829373650107991	0.7103	3
0.865931749460043	0.702475389467648	0.853131749460043	0.6955	4
0.865931749460043	0.715282423779031	0.848812095032397	0.7091	5
0.865931749460043	0.634234923062219	0.913606911447084	0.6213	6
0.865931749460043	0.686418809136959	0.883369330453564	0.6773	7
0.865931749460043	0.67533212271815	0.894168466522678	0.6652	8
0.865931749460043	0.673133900410972	0.915766738660907	0.6619	9
