# Supplementary figures and images for: Modeling the Future Distribution of Trifolium repens L. in China: A MaxEnt Approach Under Climate Change Scenarios
Source: Biology (Basel). 2025 Nov 17;14(11):1608. doi: 10.3390/biology14111608 (PMC12650469; doi:10.3390/biology14111608)

# Supplementary Material Figure

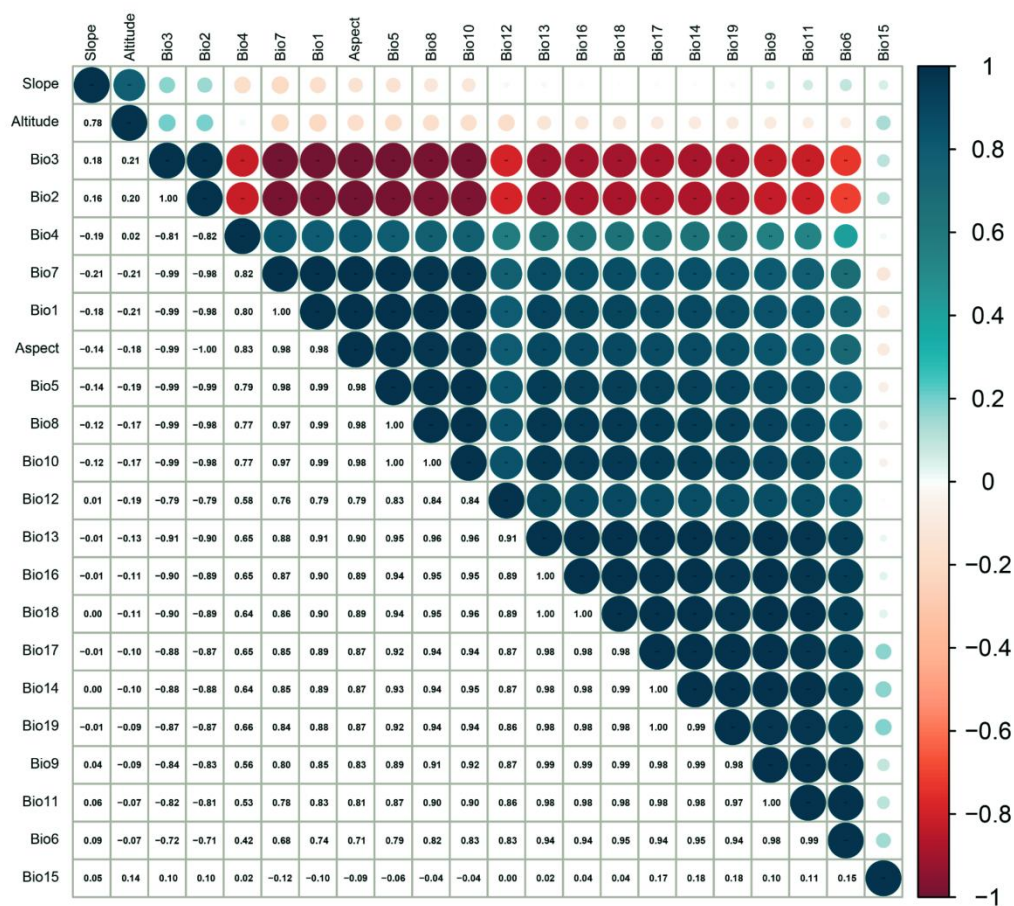

Figure. S3: Correlation Matrix Diagram of Climate Variables.

Supplement: Supplementary file 1 [file biology-14-01608-s001.zip › Supplementary Material Figure S3.pdf]

# Supplementary Material Figure

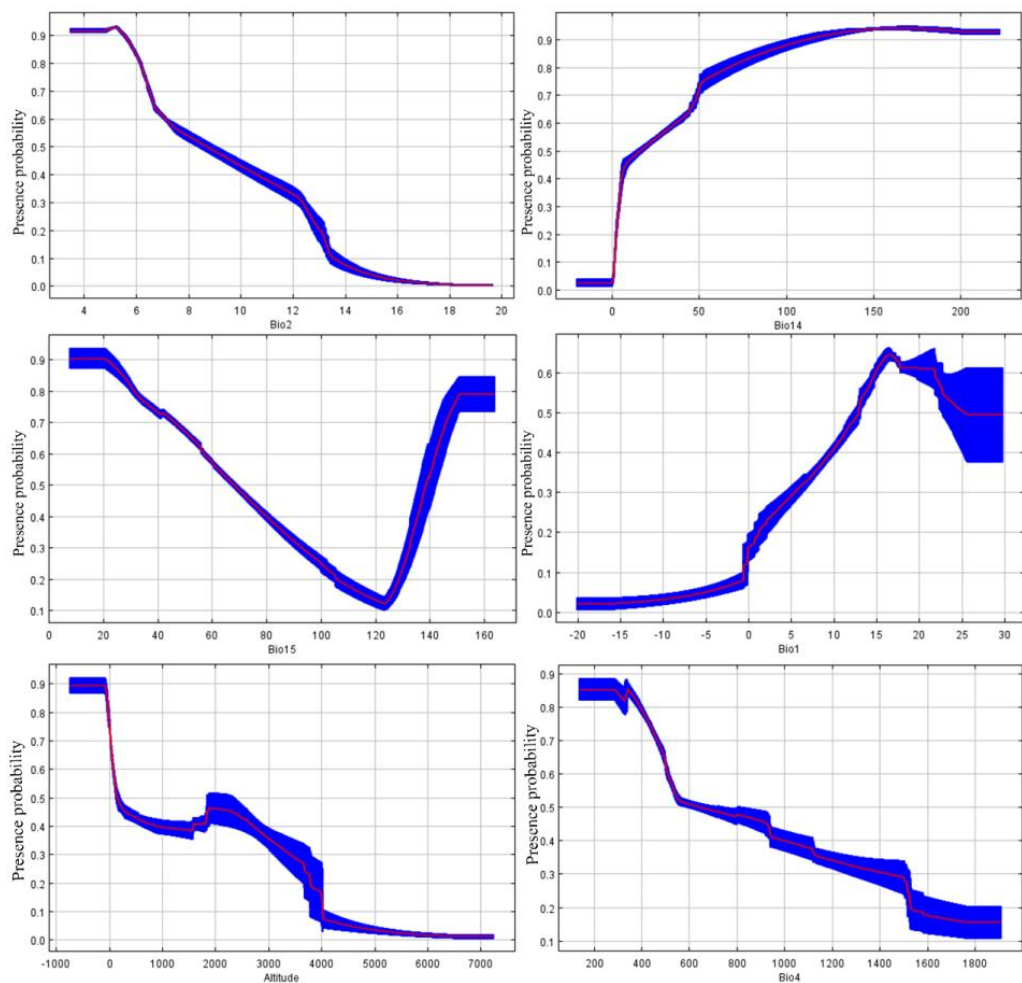

**Figure. S4:** Response curve of dominant environmental variables.

Supplement: Supplementary file 1 [file biology-14-01608-s001.zip › Supplementary Material Figure S4.pdf]
